# Large Uterine Fibroids in Pregnancy with Successful Caesarean Myomectomy

**DOI:** 10.1155/2020/8880296

**Published:** 2020-11-10

**Authors:** Edu Eyong, Okon A. Okon

**Affiliations:** Department of Obstetrics & Gynecology, Faculty of Medicine, University of Calabar, Calabar, Cross River State, Nigeria

## Abstract

Uterine fibroid is the commonest benign tumour of the female reproductive tract. It occurs in 20–40% of women, whereas the estimated incidence in pregnancy is 0.1–3.9%. Uterine fibroid in pregnancy is usually asymptomatic with complications occurring in 10–30% of cases. The first line of management is conservative with counselling for myomectomy after delivery. However, in the presence of intractable symptoms, both antepartum myomectomy and caesarean myomectomy have been reported to be successfully performed in carefully selected cases. We report a case of large subserous uterine fibroid in pregnancy that was referred to our centre at 14 weeks of gestation. She developed generalized body weakness, backache, and breathlessness at 27 weeks gestation. Thus, she was admitted and managed conservatively for eight weeks with significant relief of symptoms. She eventually had a caesarean myomectomy at 35 weeks of gestation; the outcome was a live female baby with a birth weight of 2.3 kg and a large subserous fibroid weighing 9.5 kg. We can therefore say that caesarean myomectomy can be safely performed in carefully selected cases.

## 1. Introduction

Uterine fibroids are the commonest benign tumours of the female reproductive tract which arise from the smooth muscle cells of the uterus [1, 2]. It is a common gynaecological tumour in Nigeria [3]. They could be single or multiple in number and present in varying sizes. They occur two to three times commoner in black women than in Caucasian women [1]. It occurs in 20–40% of women, whereas the estimated incidence in pregnancy is 0.1–3.9%. The much lower incidence in pregnancy could be explained by the fact that uterine fibroid is associated with infertility as well as low implantation rates after in vitro fertilization [4]. However, the incidence of uterine fibroids in pregnancy could be underestimated since most uterine fibroids are asymptomatic and a lot of women may not undergo routine ultrasound scan in pregnancy [5]. The first line of management of uterine fibroids coexisting with pregnancy is conservative with counselling for myomectomy after delivery. This is because they are usually asymptomatic with complications occurring in 10–30% of cases during pregnancy, labour, and/or puerperium [6, 7]. However, in the presence of intractable symptoms, some patients have been offered termination of pregnancy [8]. Alternatively, both antepartum myomectomy [8–10] and caesarean myomectomy [11–15] have been done successfully in carefully selected cases.

We decided to document this case of successful caesarean myomectomy done in our clinical setting of limited resources to encourage the broadening of counselling options in carefully selected cases.

## 2. Presentation of Case

A 37-year-old G3P0^+1^ lady was referred from a secondary level facility to our centre due to a huge uterine fibroid coexisting with pregnancy at 14 weeks gestation.

She presented with a seven-year history of abdominal swelling and amenorrhoea of 14 weeks duration. The abdominal swelling was progressively increasing in size with associated abdominal pain of one-month duration. There was no history of vaginal bleeding, bowel/urinary, or other symptoms.

She did not seek any medical advice or treatment for the abdominal swelling before she became pregnant. Pregnancy was confirmed by an ultrasound scan at 12 weeks gestation.

On examination, the patient was not in respiratory distress, clinically pale, afebrile, anicteric, and no pedal oedema. The pulse rate was 88 beats per minute, and blood pressure was 90/50 mmHg. The respiratory rate was 18 cycles per minute. The abdomen was grossly distended and there was a huge, firm abdominopelvic mass measuring 44 cm from the symphysis pubis. Abdominopelvic sonography showed a viable singleton intrauterine foetus of 14 weeks gestation. It also showed a huge subserous uterine fibroid measuring about 25cm × 18cm at the left anterior aspect of the uterus. Blood tests showed a haematocrit of 28% and normal electrolytes and urea and creatinine levels. The woman's blood group was O Rhesus “D” positive, and the haemoglobin genotype was AA.

The patient was seen at two weekly intervals at the antenatal clinic. She was treated with haematinics, antihelminthics, and malaria chemoprophylaxis. She was successfully treated for malaria during this period (with oral artemisinin-combined therapy) when she had clinical symptoms and a positive smear test.

She was admitted into the antenatal ward at the gestational age of 27 weeks due to complaints of generalized body weakness, back pains, and breathlessness. She had a second episode of malaria while on admission which was again successfully treated with oral artemisinin-combined therapy. She was admitted for eight weeks and was conservatively managed with bed rest, hydration, analgesics and fetomaternal monitoring. The conservative management resulted in the significant relief of all the symptoms.

During the period of admission, the patient had a transfusion of one unit of packed cells. She also received intravenous iron dextran and subcutaneous erythropoietin to correct anaemia. At the end of treatment, the haematocrit was 35%.

She was counselled for an elective caesarean section while on admission to which she gave her consent. Intramuscular dexamethasone was administered preoperatively to aid foetal lung maturity.

**Figure 1:**
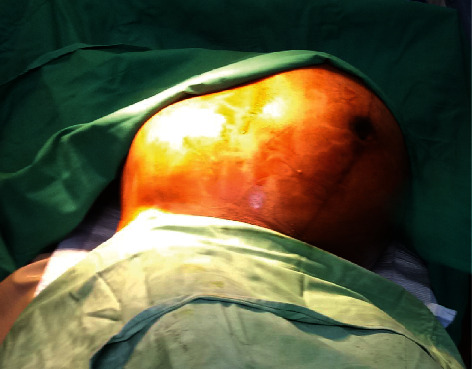
Outline of the uterus and the uterine fibroid before surgery.

**Figure 2:**
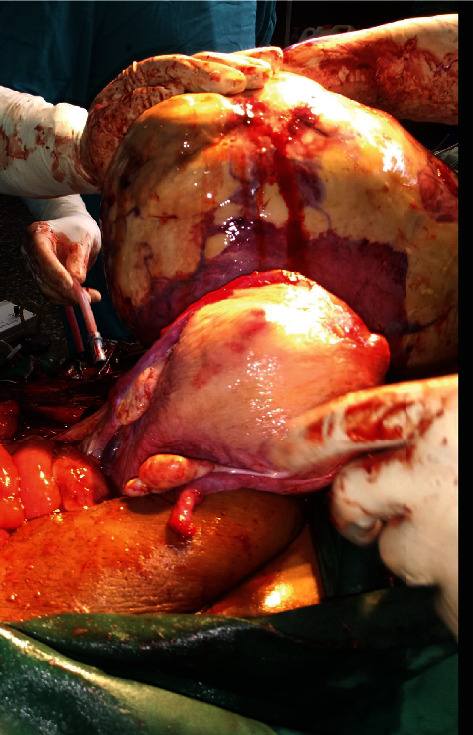
Relationship of the fibroid to the uterus.

**Figure 3:**
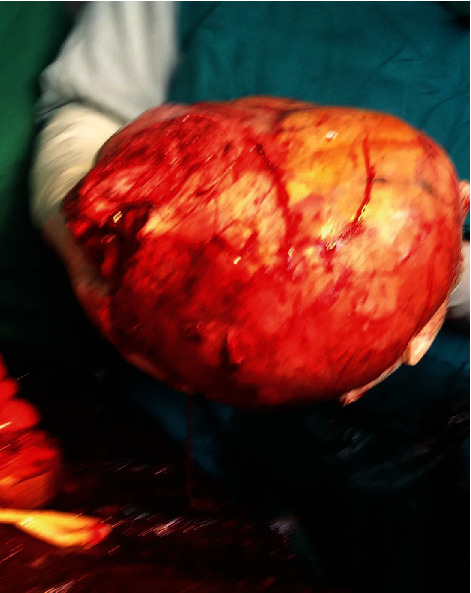
Removed fibroid nodule.

Elective caesarean section was carried out under spinal anaesthesia at 35 weeks of gestation.  Figure 1 shows the outline of the uterus and the uterine fibroid before the commencement of the operation, with the uterus deviated to the right flank and the fibroid located centrally. The abdomen was then opened by a midline subumbilical incision. The huge subserous uterine fibroid was located centrally with the uterus deviated to the right abdominal flank. The outcome was a live female infant of birth weight 2.3 kg and Apgar scores 5 and 8 at 1 and 5 minutes, respectively. Following the delivery of the baby, the uterine incision was repaired. The uterus was then exteriorized, and the relationship of the fibroid to the uterus is as shown in Figure 2. Thereafter, a Foley catheter size 18FG was applied as tourniquet at the level of the internal cervical os; intracapsular myomectomy was successfully performed with careful closure and good homeostasis ensured. The fibroid nodule as seen in Figure 3 showed areas of cystic degeneration and weighed 9.5 kg. It was sent for histology, and the report later confirmed the diagnosis of a leiomyoma with areas of cystic degeneration and no evidence of malignancy. Estimated blood loss after surgery was 800 ml, and two units of whole blood were transfused postoperatively. She also received analgesics and antibiotics postoperatively. Postoperative haematocrit was 30%. She had an uneventful postoperative period and was discharged from the hospital after five days. The postnatal visit after six weeks was satisfactory.

## 3. Discussion

The incidence of uterine fibroids in pregnancy would likely increase globally due to delay in childbearing which is more prevalent now due to different factors [16–19]. In Nigeria, like other black nations, there is a higher tendency of the women to develop uterine fibroids due to several mechanisms [16, 20]. Also, our women usually present late for treatment due to poor health-seeking behaviour, sociocultural and financial constraints, and the fear of surgery [16, 21, 22]. This delay in presentation for treatment makes our black women more likely to present with large and/or multiple uterine fibroids which have a higher risk of complications during pregnancy [7, 23].

The complications which could be associated with uterine fibroids in pregnancy include degenerative changes which commonly cause abdominal pain, miscarriages, malposition/malpresentation, intrauterine growth restriction, antepartum haemorrhage, preterm labour, obstructed labour, postpartum haemorrhage, and high caesarean section rates [16–19, 24–26].

The occurrence of some of these complications in pregnancy could lead to the abandonment of the usual conservative management of uterine fibroid in pregnancy. The option of myomectomy during pregnancy must be carefully considered. There is much increased uterine vascularization during pregnancy. Therefore, myomectomy may result in excessive blood loss which may lead to inevitable hysterectomy [5, 23] or maternal mortality [16]. In the presence of intractable symptomatology, however, we encourage treatment to be individualized to get optimal results.

Our index patient noticed progressive abdominal swelling over seven years but did not present for treatment in any hospital before she got pregnant. She had responded well to conservative management; thus, antepartum myomectomy was not performed. Despite comprehensive counselling, we had no way of ensuring that the patient would return for myomectomy after the delivery of the baby. Also, the lady was primiparous; thus, she would likely have returned with more complications due to uterine fibroids in subsequent pregnancies. Caesarean myomectomy was further adjudged to be safe in this case because the ultrasound scan showed a single, large, subserous fibroid nodule. Based upon the above considerations, an intracapsular caesarean myomectomy was successfully performed after delivery of the baby. This is the currently recommended technique for caesarean myomectomy [27, 28] which is safe, feasible, and reliable if correctly performed in carefully selected patients [27–29]. Intracapsular myomectomy technique is currently recommended because the fibroid pseudocapsule (which is preserved during the procedure) has been shown to contain many neuropeptides and neurotransmitters. These substances play a positive role in wound healing and improvement in subsequent sexual and reproductive functions [30]. Our index patient is nulliparous and thus would benefit from this surgical technique. Thereafter, she had no postoperative complications due to good preoperative preparations, good surgical skills, and good postpartum monitoring which anticipated and prevented complications.

We decided to document our experience in our clinical setting where many patients may present with large and/or symptomatic uterine fibroids in pregnancy. This goes to show that caesarean myomectomy could be performed with good results in carefully selected cases.

## 4. Conclusion

Uterine fibroids in pregnancy are usually asymptomatic, and thus, the first line of management is conservative. However, in the presence of complications, good selection criteria need to be applied to individualize patient care for optimal results. Caesarean myomectomy can be performed with good results in carefully selected cases, as was obtained in this case report.

## Data Availability

Availability of data is not required.
